# Preliminary Study on the Capability of the Novel Near Solidus Forming (NSF) Technology to Manufacture Complex Steel Components

**DOI:** 10.3390/ma13204682

**Published:** 2020-10-21

**Authors:** Gorka Plata, Jokin Lozares, Andrea Sánchez, Iñaki Hurtado, Carl Slater

**Affiliations:** 1 Mechanical and Manufacturing Department, Mondragon Unibertsitatea, Loramendi 4, 20500 Mondragon, Spain; jlozares@mondragon.edu (J.L.); asanchezfe@mondragon.edu (A.S.); ihurtado@mondragon.edu (I.H.); 2 WMG, University of Warwick, Coventry CV4 7AL, UK; c.d.slater@warwick.ac.uk

**Keywords:** steel, metal forming, Near Solidus Forming, complex geometries

## Abstract

The benefits of the novel Near Solidus Forming (NSF) process has shown previously in its ability to produce steel components with comparable as-forged mechanical properties but with a cost reduction of 10–15%. This study further pushes the NSF technology to produce parts that are conventionally difficult to produce via conventional methods. A 2.7 kg 42CrMo4 steel grade component was manufactured into a complex geometry using only a 400t press. Different manufacturing parameters were evaluated to show their influence on the process and final component. A combination of X-ray fluorescence (XRF), optical microscopy and SEM analysis of the microstructure was also conducted revealing the deformation pattern of the material and shedding some light on how the material evolves during the process. The successful forging of these components shows the capability to produce previously deemed difficult geometries, with much a lower specification forging press, in a single deformation.

## 1. Introduction

Hot processes can be separated into either hot forging-like processes or casting-like processes. The selection of one or another lies on the final characteristics of the parts. Whilst hot forging-like processes ensure great mechanical properties of the components, casting-like processes are capable of attaining geometries that are more complex. Inversely, forging-like processes make it difficult and even impossible to attain complex geometries (without post machining the component); at the same time the cast components do not benefit from the grain refinement that occurs during recrystallisation, and therefore typically have reduced mechanical properties. There is, therefore, a balance that needs to be struck between shape and properties when choosing between these techniques.

In this sense, great effort was put into Semi-Solid Processing (SSP) technology since the discovery of the thixotropic behavior on metals in the early 1970’s [1]. This phenomenon enabled a liquid-like behavior with solid particles already present inside [2]. This opened up the potential of attaining very complex geometries (as with casting) while improving the components’ mechanical properties. Most of the investigations were focused on low solid fractions and low melting point alloys, where very good results were attained and some processes are actually industrialized [3,4,5].

Due to the low solid fractions, the mechanical properties of SSP were still not comparable to those obtained in bulk deformation processes with some casting defects such as porosity still being present. Moreover, as those processes were based on machines similar to those used for High Pressure Die Casting (HPDC), implementation of this technology using high melting point alloys became difficult (although some investigations were carried out [6,7]).

Consequently, in an attempt to achieve mechanical properties more comparable to that of forging as well as being applicable for high melting point alloys, high solid fractions began to be used with steels in the 1990’s [8]. From then on, several authors have assessed the manufacturing of different complex geometries [9,10] and even special alloys were manufactured to enhance the SSP working window and reduce the working temperature [11]. However, they observed premature die degradation, which among others, hindered the possible industrialization [12,13].

These problems have been proposed to be addressed through using Near Solidus Forging (NSF) technology. Previous works assessed how the manufacturing of large batches of components without observing any severe die damage has been possible, attaining as-forged properties [14,15,16], using off-the-shelf alloys and reducing by nearly 10 times the force required to fabricate the component by conventional hot forging [17]. This mainly comes from the optimization of the working conditions and equipment. In this sense, the material is no longer in the classical SSP conditions. The material is heated up to temperatures below the steel solidus temperature and then deformed in a press in closed die system. This produces a part that has comparable mechanical properties to conventional forging with an ultimate tensile strength of 1073 MPa compared to the 1030–1130 MPa required for 42CrMo4 [17]. According to the best of the author’s knowledge, at the moment, there are no other studies on processes at the same conditions as NSF. The closest studies are some SSP at very high solid fractions [18].

The NSF process therefore certainly offers the benefits to mechanical properties whilst also utilizing much lower specification forging presses (400t compared to 3000–4000t in hot forging). This, together with the material consumption reduction due to the avoidance of flash generation and the possibility to reduce the machining allowances, permits the attainment of a cost reduction of the order of 10–15% [17].

Nevertheless, the limitations regarding the processing capabilities of the NSF process are still unknown. This means that it is still not known how to re-design a component in order to take the highest advantage of the NSF process. Aspects such as achievable minimum thicknesses or maximum length are still undetermined. This study will therefore address the geometric aspect of NSF and aims to form a component that is otherwise “impossible” to produce via conventional forging means.

## 2. Materials and Methods

### 2.1. Geometry

**Figure 1:**
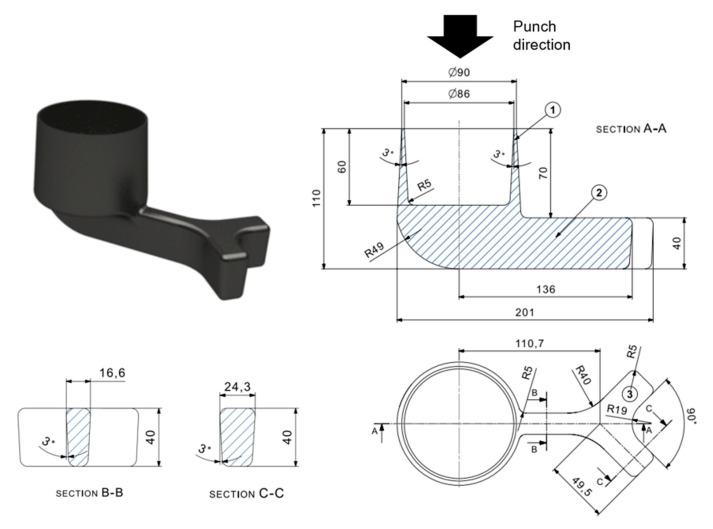
New component design to test the filling capabilities of the material during the Near Solidus Forming (NSF) process.

Considering all the above, a new geometry design was developed in an attempt to discover the limitations the material has to filling certain complex geometries. As can be observed in the following figure (Figure 1), the geometry consists of an upper cup shape of thin walls attached to a long arm.

The geometry (Figure 1) consists of an upper cup of 60 mm height with thicknesses ranging from 10 mm (in the bottom part) to 2 mm (in the upper part) flowing against the press direction (zone 1 in Figure 1). The diameter of the cup is 90 mm on the upper side and 97 mm on the bottom. The cantilever arm was added below the cup to account for the limitations to fill long distances (zone 2 in Figure 1). This arm is around 150 mm long (from the center of the cup) with an initial section of around 16 mm thick and 40 mm height (section B-B in Figure 1). In the last part of the arm, two fingers of bigger cross-section (24 mm thick and 40 mm height as shown in section C-C in Figure 1) were also designed (zone 3 in Figure 1) to check how the material behaves under a negative cross-section change (from a smaller to bigger cross-section).

The filling of this geometry is therefore challenging due to three complex zones (as labelled in Figure 1). As mentioned before, the cup area (Zone 1) is particularly challenging as it is designed to test the minimum thicknesses that can be filled by NSF in the cell of Mondragon University, with flow in the opposing direction to the ram. Zone 2, has a 90° change in the filling direction, a feature that is rarely obtained in the forging industry. Lastly, the negative cross-sectional change of the fingers will enable the analysis how the material evolves with a sudden increase in cross-section which also requires a splitting of the flow material.

### 2.2. Material

In this preliminary work, where the limits of the process in terms of filling capabilities are sought, the selected steel grade is the well-known 42CrMo4, a quenching and tempering medium carbon steel alloy generally used in aeronautic and automotive industries. This material was one of the first materials used in NSF obtaining great results and, hence, its forming conditions are better defined than with other materials. Moreover, this steel ensures the attainment of as-forged properties after the post-processing quenching and tempering heat treatment as stated in [14]. The following table (Table 1) shows the chemical composition of the alloy.

### 2.3. NSF Cell

**Figure 2:**
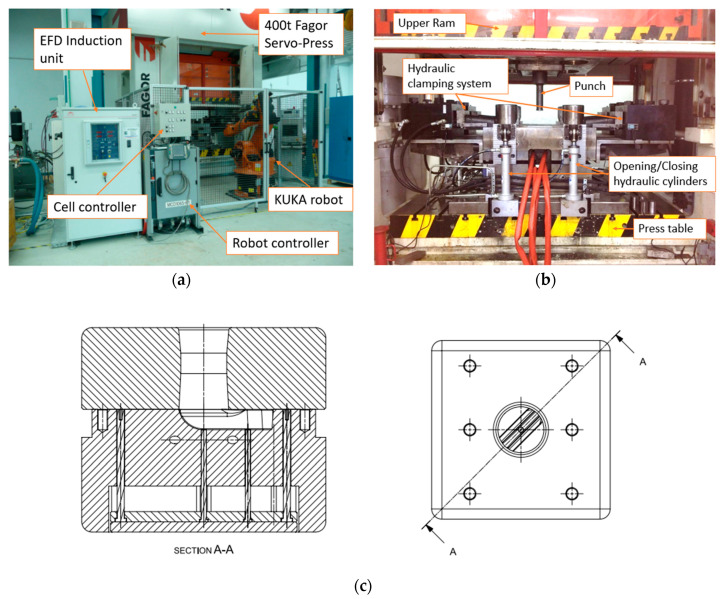
(**a**) The NSF cell of Mondragon University, (**b**) the self-designed NSF tooling for high melting point alloys [17] and (**c**) schematic drawing of the dies and expulsion system.

The NSF cell in Mondragon University consists of an EFD induction furnace, a six-axis KUKA robot with a self-designed grip and a Fagor 400t alternating current (AC) servo-mechanical press (Figure 2a). The deformation tooling that goes into the press was also self-designed, consisting of two-die closing die system (Figure 2c) attached to the press table and an injection punch attached to the ram (Figure 2b). For a more detailed description of the cell go to [17].

### 2.4. NSF Process

#### 2.4.1. Initial Preparations

At this stage, the dies are pre-heated to around 250 °C–300 °C with the flow of oil through the tempering channel of the dies. Then, CeraSpray^®^ long-lasting ceramic varnish with lubricant effect that acts also as a thermal shock barrier is applied in both dies and punch (as suggested by Pierret et al. [19]). Then, the dies are closed and clamped.

The final geometry has a volume of around 346,000 mm^3^ and so a standard starting billet of 70 mm of diameter and a 90 mm height of the 42CrMo4 were used.

#### 2.4.2. Heating of the Billet

**Figure 3:**
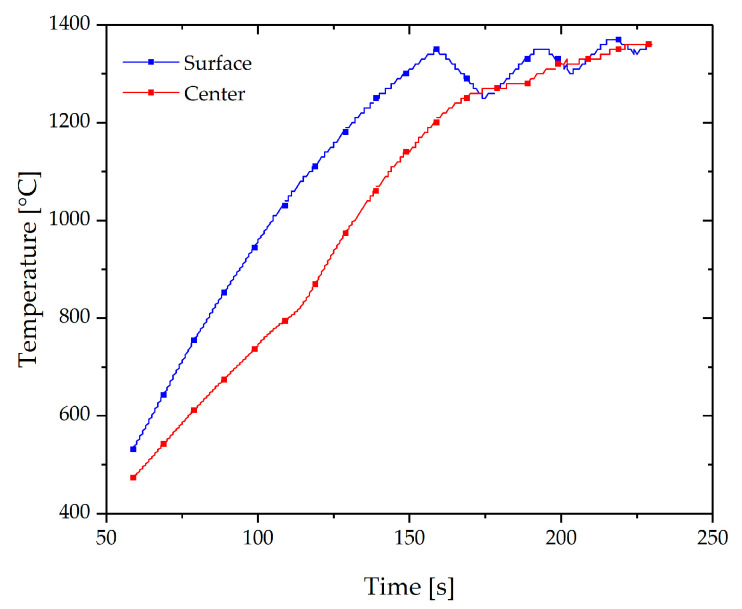
Example of the recorded temperature evolution in the center and surface of the billet during pulse heating for Trial 1.

For heating a 150 kW induction furnace was then used. Due to the nature of heating, there is an unavoidable inhomogeneous temperature difference between the surface and the center of the billet (skin effect). Consequently, a several stage heating cycle is commonly used to overcome this problem. In this case, a cycle consisting of four heating stages was proved to be suitable to reduce that inhomogeneity [20]. This briefly consists of pulse heating at 25 kW for 3 min and 50 s (for Trial 1) to achieve a homogeneous temperature through the billet (Figure 3). The solidus temperature of this material is around 1410 °C.

During heating an argon protective shield was used at a flow rate of 14–20 L/min to avoid the generation of oxide in the surface of the billet.

#### 2.4.3. Transfer Stage

Once the heating of the billet is finished, a robotic arm lifts and places the billet onto the press. To reduce time then the robotic arm is moving fast and the transfer time is about 12–14 s. This process is automated and therefore consistent. Typically, the billet cools by around 20 °C–30 °C during this period.

#### 2.4.4. Deformation Stage

**Figure 4:**
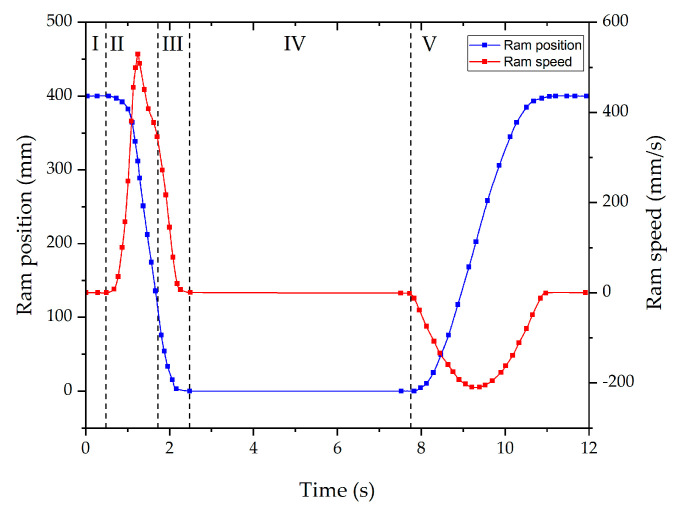
Schematic representation of the default press position and speed during NSF process: **I**—press waits for the billet placement, **II**—punch approaching the billet, **III**—Deformation stage, **IV**—5 s of dwelling at bottom dead center (BDC) and **V**—ram movement back to top dead center (TDC).

The deformation stage consists of moving the punch from the top dead center (TDC) to the bottom dead center (BDC) of the ram (stage II in Figure 4 means the approaching stage and stage III in Figure 4 means the deformation stage). The ram position is defined considering that the stroke of the press is 400 mm and that the maximum load is only attained at the last displaced distances. The displacement is maintained at the BDC dwelling for 5 s (stage IV in Figure 4). An example of the cycle is shown in Figure 4.

**Figure 5:**
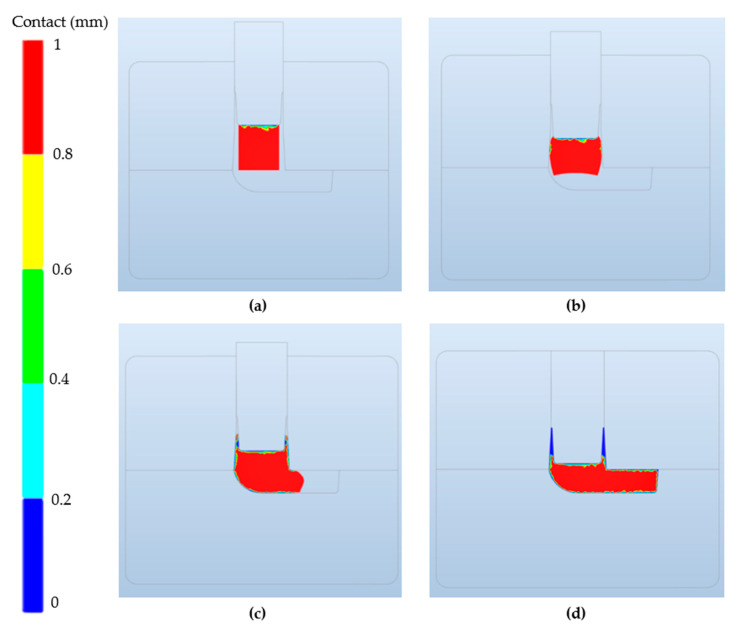
Component filling illustration at different punch positions during the NSF process (Stage III in Figure 4) using FORGE^®^ simulation software. (**a**) Initial position, (**b**) 46 mm punch displacement, (**c**) 62 mm punch displacement and (**d**) complete filling. The blue color means contact with the dies/punch. The red color shows those areas that are more than 0.8 mm distance from dies and punch.

Because of the ram deceleration when approaching the BDC, the speed of the punch when it strikes the billet is around 350 mm/s. An example of the filling sequence of the selected geometry for this research work is shown in Figure 5.

#### 2.4.5. Component Ejection

To eject the component, once the deformation stage has finished and the ram is at the TDC (stage IV in Figure 4), the clamping system retraction and die opening is manually activated. Once the dies are fully separated, the pneumatic cylinder of the press is manually activated to eject the component. Then, an operator is the responsible for retiring the component from the press and cleaning the dies.

### 2.5. Microstructural Analysis

Different equipment was used for the microstructural evaluation of the post processed components. To begin with, a slice from the middle of the component was introduced in a Bruker Tornado M4 X-ray fluorescence (XRF) system to compositionally map the component. The evaluation set up consists of a 100 μm step size with a 100 ms dwell time on each point. The gathered information at this point will show how the component state is internally at a macro scale level. Moreover, different micro hardness tests were carried out through different zones of the slice using an AFRI^®^ 206 EX hardness tester to also observe hardness variability. The XRF and hardness information will be then the base to define the most interesting areas for the subsequent micro scale analysis.

Regarding the micro level analysis of the material structure, several steps were performed. Fist, the samples were prepared by grinding, polishing and etching with 2% Nital. Then, the microstructures were analyzed using a Leica DMC2900 optical microscope and an FEI NovaNanoSEM 450 Scanning Electron Microscope (SEM) equipped with an Oxford X-max 50 X-ray detector (EDX). With the combination of both optical microscopy and SEM, the most interesting zones of the component according to the XRF results were evaluated in more detail.

### 2.6. Experimental Sequence

The conditions of the conducted experiments are listed in the table below (Table 2). As observed, different temperatures and billet sizes have were in an attempt to observe their influence on the filling capabilities of the material during the NSF process. As the material temperature changes, the heating cycle is also modified to achieve a homogeneous temperature through the billet. The rest of the processing parameters are the same for each trial.

## 3. Results and Discussion

### 3.1. Component Manufacturing

**Figure 6:**
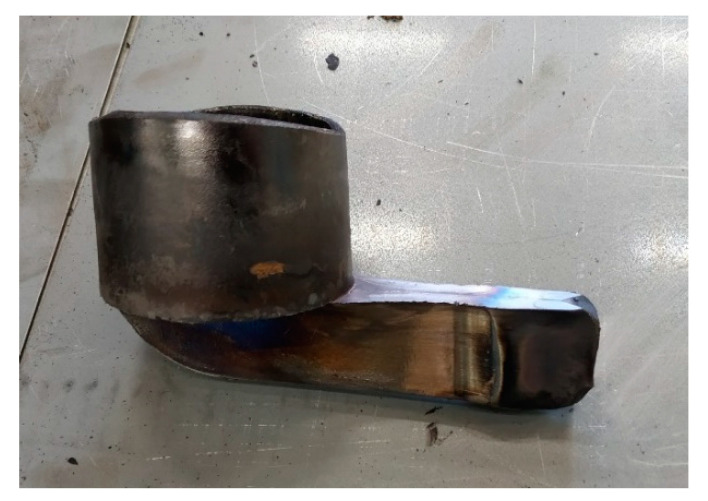
Obtained component after the first NSF trial with the 42CrMo4.

**Figure 7:**
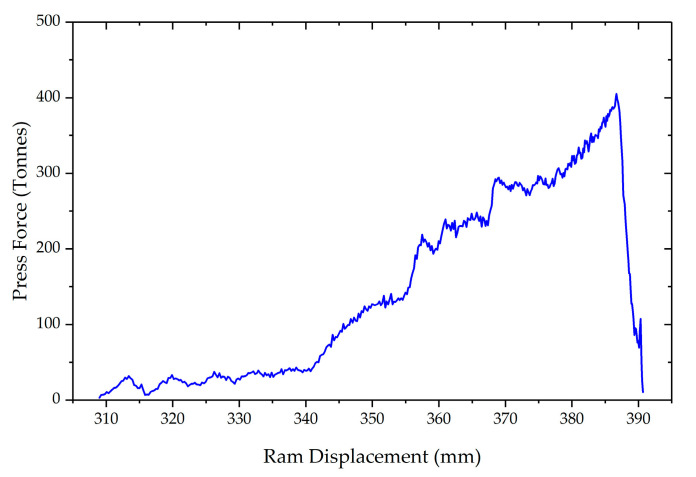
Example of the recorded press force against the displacement of the press for Trial 1.

For the very first trial, the conditions at which sound components were attained in the past with other geometries were used [14]. Therefore, a forging temperature of 1360 °C was used with an initial billet size of 70 mm of diameter and 90 mm height. This trial reached the load limit of the cell (380t to protect the 400t machine) before deformation was complete. The obtained component can be seen in Figure 6. Both the cup and arm can be seen to have only partially filled (around 79% of the total volume). Figure 7 shows the ram force against displacement for this trial (corresponding to the stage III of the process explained in Figure 4) and the abrupt stop of the press when the soft limit overloading was detected (being incapable of completely filling the cavity).

There are multiple reasons why underfilling can occur, however this geometry requires a right angle turn, the load is normal to the arm direction and opposite to the cup filling direction, and therefore the requirements are much more than a simple unidirectional forging operation. To help overcome this two parameters can then be modified to check its influence: billet temperature and billet diameter. In this case, a higher temperature and bigger billet diameter (maintaining the same volume) were tried separately whilst maintaining the forming cycle described in Figure 4. Trial 2 consisted of a higher temperature of approximately 1390 °C but maintaining billet geometry. Trial 3 used the same temperature for Trial 2 but increased the billet diameter to 75 mm and height to 79 mm.

**Figure 8:**
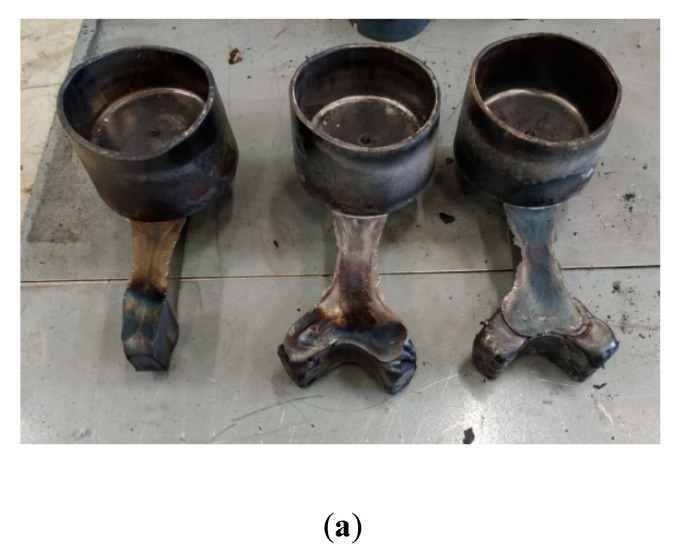

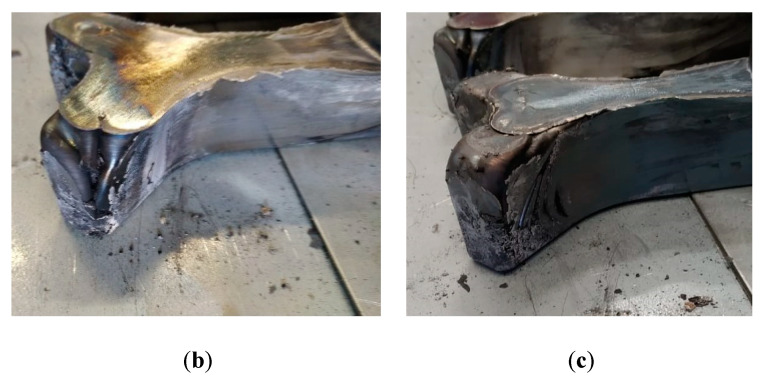
(**a**) Obtained components in Trials 1, 2 and 3, respectively, (from left to right). Detail of the last part filling (fingers) in (**b**) Trial 2 and (**c**) Trial 3.

**Figure 9:**
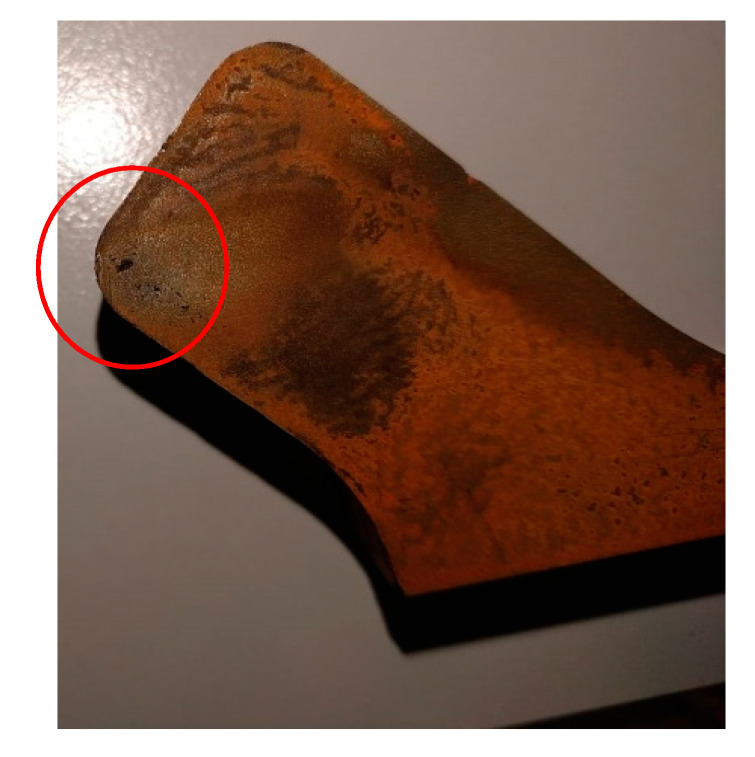
Defects located at the very end of the last filling regions (unfilled regions) of Trial 3.

The conditions of Trial 2 resulted in a larger degree of filling compared to Trial 1, with Trial 3 showing almost complete filling of the cavity (Figure 8). However, none of these cases showed complete filling of the die. Regarding the cup area, thicknesses of the order of 3 mm were attained. Thicknesses such as this are very difficult during conventional forging, as this would require multiple strikes, and cooling in these regions would be rapid and soon “freeze” in the mold, preventing further flow. From Figure 9 it can be seen that the components are, for the majority, defect free, however a small number of defects can be seen in the final corner.

**Figure 10:**
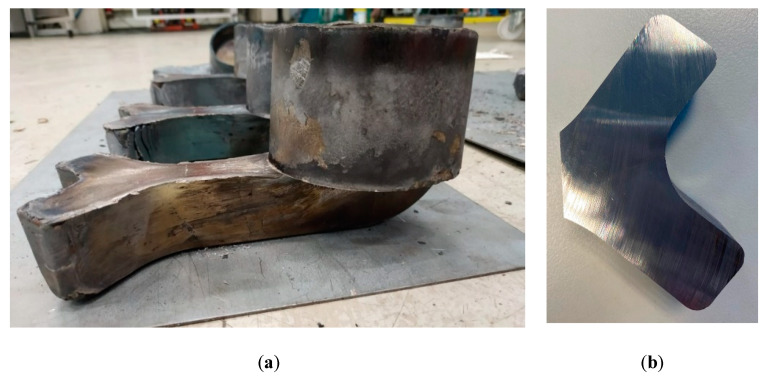
(**a**) Obtained component with the arm completely filled and (**b**) slice of the last filled regions.

In a final trial (Trial 4) a billet of 85 mm in diameter and 61 mm in height was heated to 1380 °C–1390 °C, the soft limits were removed and the NSF press achieved 400t load. This component can be seen in Figure 10. This component showed complete filling of the arm with cup section filling to around 98.45% and with a wall thickness of 2.8 mm.

These results definitely confirm how some parameters can imply a huge difference in terms of filling capability of the material and, also, the flexibility of the NSF to adjust the best forming parameters and conditions depending on the geometry in order to make the process as cost-effective as possible. In this case, the temperature was increased (to enhance the flowing capabilities of the material) at levels where, in theory, the component can be filled without generating liquid-related defects.

Moreover, the defects observed in Figure 9 observably disappeared. Even if those defects are flow folds closing or porosity, it is evident how these are a result of an under-deformed component and that with the correct load then this feature will be removed. Further work will look into these features as this will help to drive whether lubrication (to reduce folds), or by introducing channels in the die (to allow gas to escape), will aid the production moving forward.

**Figure 11:**
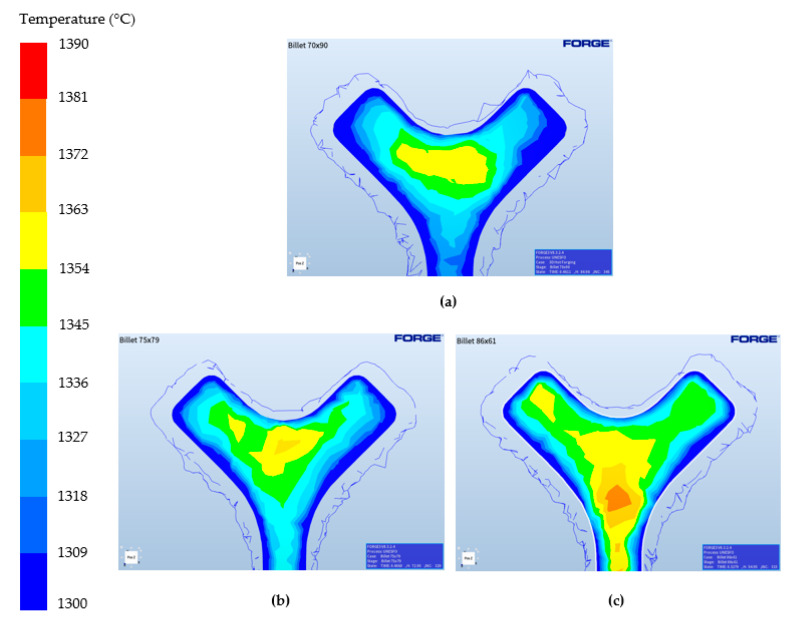
FORGE^®^ simulation results showing the temperature of the component in the last stages of deformation for different initial billet dimensions (in mm): (**a**) ø70 × 90, (**b**) ø75 × 79 and (**c**) ø85 × 61.

Regarding the effect of the billet, the bigger the diameter (and smaller the height) the better the filling capabilities. This mainly comes from the fact that, at lower billet heights, the contact time with the punch decreases because it needs shorter ram displacements to completely fill the cavity. Hence, due to the lower pressurized contact time with the punch, the temperature of the billet remains higher compared to larger billets (Figure 11). This therefore implies a better deformability in the latter deformation stages due to the higher temperatures.

### 3.2. Microstructural Evaluation

**Figure 12:**
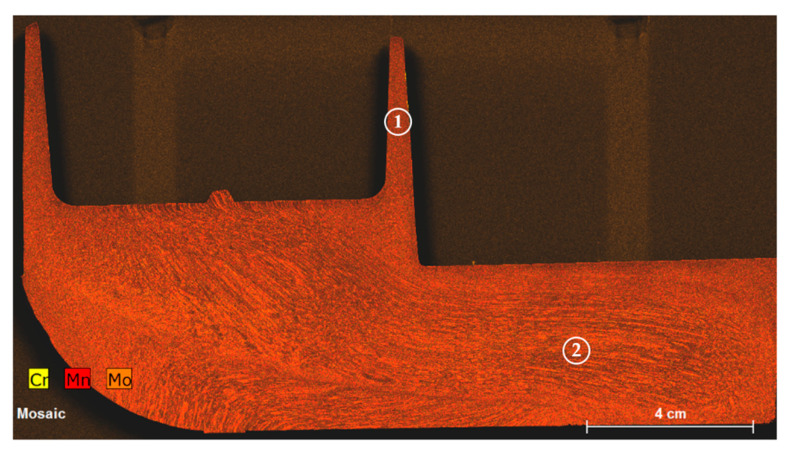
X-ray fluorescence (XRF) analysis of the slice taken from the component manufactured with the 42CrMo4. Zone 1 and 2 are the cup and bulk regions respectively.

After observing the successful results at a geometric level, it is necessary to confirm that the components are also sound internally. The XRF map of Cr, Mn and Mo can be observed in Figure 12. Due to the composition of this steel and the segregation these elements show, then we can see the flow behavior of the steel to achieve the component geometry. After the billet hits the bottom of the die then a shearing of the billet occurs to translate the vertical load into a horizontal displacement, after which laminate flow can be seen to continue to fill the arm. The cup region shows very little flow pattern suggesting the strain in the region is much higher, as to be expected. No porosity was found throughout this section of the component.

**Figure 13:**
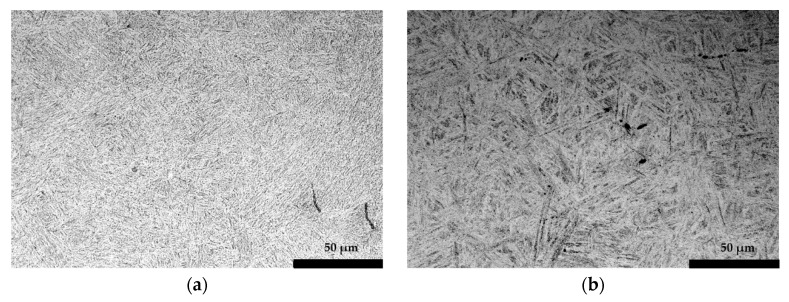
Microstructures taken from (**a**) the cup and (**b**) the bulk areas of the component.

The microstructure and phases present in the component are shown in Figure 13. The microstructure is quite homogeneous through the component consisting on tempered martensite with some areas of lower bainite in bulk regions. This means that the cooling differences are minor in the component, being almost the whole part capable of attaining the martensitic structure and with little differences in tempering. This is confirmed by the obtained hardness values, which were consistently 105–115 HRB in all areas.

**Figure 14:**
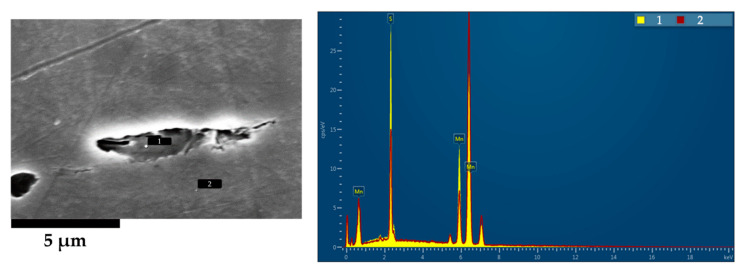
Compositional analysis of the observed particles in the FEI NovaNanoSEM 450 Scanning Electron Microscope (SEM) equipped with an Oxford X-max 50 X-ray detector (EDX).

Figure 14 shows the presence of MnS inclusions; these are purposeful addition to the composition to aid in the post machining of forged components. Due to the low hardness of MnS they deform easily and show the flow behavior of the material. The MnS is stable up to 1420 °C in this steel (as calculated by Thermo-Calc 2020b TCFe10) and therefore does not dissolve during reheating and is present during deformation.

The presence of these particles during deformation can be used as a tracker of the amount of deformation in different zones. As observed in Figure 13, the shape of these inclusions is quite different depending on the location, being more elongated in the cup zone (Figure 13a) than in the bulk (Figure 13b). The aspect ratio (understanding it as the division between larger length of the inclusion with the shorter one) of the MnS therefore gives an indication of the level of strain in the related areas. The aspect ratio in the cup is 10 while in bulk areas it is around 3. However, in the initial as-received material they showed an aspect ratio of 1–2. This suggest that at the core very minimal strain occurs (≈0.67 true strain) whereas in the cup region a true strain >1.2 is shown. True strain was calculated from the below equation (Equation (1)), assuming unity of the initial:

(1) $$\epsilon =LN\left(\frac{1}{\sqrt{Aspect\;Ratio}}\right)$$

This high strain in the thin section is critical, for flow to occur maintaining a high enough temperature so that dynamic recovery can take place for minimal work hardening is essential. What is also key is that even though a small amount of strain is taking place in the core, this is still typically high enough to show dynamic recrystallisation and refine the grains, without which this region would show a large grain size due to the excessive grain growth at these temperatures (with the consistent hardness showing that this is not the case).

## 4. Conclusions

In this work, the NSF processing capabilities were tested with a new complex geometry design. The conducted experiments are preliminary tests to shed some light on the NSF processing limits in terms of material deformation capabilities. Hence, the results of this work should be taken as an indicative approach to what can be obtained in the NSF process and the possible influencing parameters.

All in all, this work has widened the knowledge regarding the NSF of steels by confirming the following:

- Thicknesses below 3 mm can be achieved using the 42CrMo4 medium carbon alloy.
- Long distances perpendicular to the forming direction can be successfully filled.
- The material can fill negative cross-sections (from smaller to bigger cross-sections).
- The process enables the closure of defects such as folds and pores.
- The NSF process generates strain vectors in a single deformation that are otherwise difficult to attain via conventional forging routes.

The NSF process still needs much more experimentation and research to completely understand and define the processing limits in terms of material and processing conditions. In any case, what is clear is that the NSF process is capable of achieving sound components of very complex shapes requiring low forming loads and, as assessed in previous works [14,15,16], as forged mechanical properties whilst reducing the component cost compared to the hot forged one by around a 10–15%.

## Figures and Tables

**Table 1:** Chemical composition of the 42CrMo4 medium carbon steel (in wt.%).

C	Mn	Si	P	S	Cr	Ni	Mo
0.42	0.80	0.25	0.011	0.024	1.08	0.10	0.21

**Table 2:** Summary of the processing parameters for the conducted experiments.

	Billet T (°C)	Billet Size (mm)	Die T (°C)	Argon (dm^3^/min)	Heating Cycle (Pulses/Heating Time)	Lubricant
**Trial 1**	1360	ø70 × 90	270	14–20	4/230″	CeraSpray^®^
**Trial 2**	1390				5/258″	
**Trial 3**		ø75 × 79				
**Trial 4**		ø85 × 61				
